# Investigation of the Dynamic Breakdown of a Dielectric Elastomer Actuator Under Cyclic Voltage Excitation

**DOI:** 10.3389/frobt.2021.672154

**Published:** 2021-04-21

**Authors:** Xuejing Liu, Yu Xing, Wenjie Sun, Zhouqiang Zhang, Shengqi Guan, Bo Li

**Affiliations:** ^1^College of Mechanical and Electrical Engineering, Xi'an Polytechnic University, Xi'an, China; ^2^School of Mechanical and Precision Instrument Engineering, Xi'an University of Technology, Xi'an, China; ^3^Shaanxi Key Laboratory of Intelligent Robots, School of Mechanical Engineering, Xi'an Jiaotong University, Xi'an, China

**Keywords:** dielectric elastomer, cyclic excitation, dynamic breakdown, breakdown voltage, maximum cycle number

## Abstract

The dielectric elastomer (DE) is a new kind of functional polymer that can be used as a smart actuator due to the large deformation induced by voltage excitation. Dielectric elastomer actuators (DEAs) are usually excited by dynamic voltages to generate alternating motions. DEAs are prone to premature breakdown failure during the dynamic excitation, while the research on the breakdown of DEAs under cyclic voltage excitation is still not fully revealed. In this paper, the dynamic breakdown behaviors of DEAs made from VHB4910 film were experimentally investigated. The factors affecting the breakdown behavior of DEAs under dynamic voltages were determined, and the relevant changing laws were summarized accordingly. The experimental results show that under dynamic voltage excitation, the critical breakdown voltage of DEAs were augmented slowly with voltage frequency and showed a substantial dispersion. In addition, the maximum cycle numbers before breakdown were significantly affected by voltage parameters (such as frequency, amplitude, waveform). Finally, the underlying mechanisms of breakdown under cyclic voltages were discussed qualitatively, a power-law equation was proposed to characterize the maximum cycle number for the dynamic breakdown of DEAs, and related parameters were fitted. This study provides a new path to predict the service life of DEAs under dynamic voltage.

## Introduction

Soft robots with compliant bodies have become a research hotspot in recent years, and different kinds of soft smart robots and robotic machines have been designed and optimized (Alatorre Troncoso et al., 2018; Nan et al., 2018, 2019; Wang et al., 2018; Dong et al., 2019). Electro-active polymers (EAPs), as a potential smart material that can be utilized in the soft robot's actuator, have attracted much academic attention due to extraordinary high deformability and flexibility (Baughman, 2003; Carpi et al., 2010; Ma et al., 2013). According to the deform mechanism, EAPs can be divided into two categories depending on the activation mechanism. One category is ionic EAPs; polymers will exhibit bending deformation due to the ionic migration inside the porous film during actuation. The other category is electronic EAPs, which can induce large displacement while activated under a high voltage. Dielectric elastomer (DE), as a main branch of electronic EAPs, received a great amount of attention due to their properties of large deformation, fast response, and high electromechanical conversion efficiency.

In view of the mechanical performances, DE has been applied to novel soft actuators and structures by various researchers (Pei et al., 2004; Loverich et al., 2006; Bauer, 2013; Chen et al., 2019). Although DE has good application potential in soft structures, mature soft actuator products based on DE are rarely seen, and most of them are still in the experimental stage. The reason lies in the low reliability and its proneness to instability and breakdown during the actuation process (Jean-Sébastien and Steven, 2006). Different from rigid dielectrics with a fixed value of breakdown strength, DE is actuated under the electromechanical coupling process. The traditional breakdown model is no longer suitable for DE, and a new approach to predict the breakdown strength for DE is thus imperative. Until now, investigations into the breakdown behavior of DE have been concentrated on the quasi-static excitation situation, where the critical breakdown voltages can be predicted properly under different prestretch ratios and ambient temperatures accordingly (Huang et al., 2012; Liu et al., 2017a,b). However, dielectric elastomer actuators (DEAs) are always subject to cyclic voltage excitation to realize alternating deformation in actual applications (Loverich et al., 2006; Zhu et al., 2010; Hosoya et al., 2015), such as micro-pumps, and vibration in structures. DEAs are prone to premature breakdown failure during the dynamic actuation, yet the systematic research on the breakdown of DEAs under cyclic voltage excitation still remains rare.

Stoyanov et al. studied the fatigue behavior of DE under cyclic mechanic loading and found out that the fatigue life of DE is around 10^7^ times (Stoyanov et al., 2017), whereas, in the investigation of the DE-based pumps, it was clear that the life appeared far less than 10^7^ due to the occurrence of premature breakdown. Li et al. (Chen et al., 2017) established a theoretical model to simulate the deformation response of DEA in a pure shear mode under sinusoidal voltage excitation. It can be concluded that the dynamic deformation response is significantly influenced by affecting factors such as frequency and amplitude. Matysek et al. (2011) investigated the lifetime of dielectric elastomer stack actuators made of silicone, which is affected by various factors, such as frequency and electrode materials.

Chen and Wang (2015); Chen et al. (2015) theoretically investigated the dynamic response of the balloon subject to a combination of pressure and periodic voltage. For the first time, they concluded that the balloon is at its most susceptible to dynamic electromechanical instability, such as snap-through and snap-back, when the superharmonic, harmonic, or subharmonic resonance is excited. de Saint-Aubin et al. (2018) experimentally studied the electromechanical aging of DEA with different carbon-based electrodes, the results show that the aging in electrode resistance and actuation strain mainly depends on the total accumulated time. Yi et al. (2019) recently conducted a preliminary experiment to research the fatigue life performance of silicone-based DEAs under square wave voltage, and they found that early failures occur near the edge of electrodes. It could be possible that DEAs can last much longer if the electrodes are patterned properly.

In the present work, experimental data on the dynamic breakdown behavior of circular DEAs made of VHB 4910 film were presented for the first time. Major influence factors for dynamic breakdown behavior of DEAs were recognized through experiments, and relevant changing rules were summarized. By comparing the experimental results of dynamic breakdown with quasi-static breakdown, we found that breakdown voltages under cyclic voltage excitation increased slowly and showed a large dispersity. In addition, the maximum cycle numbers were tightly related to voltage parameters, such as frequency, amplitude, and waveform. A power-law empirical equation was utilized to characterize the maximum cycle number for the dynamic breakdown of DEAs for a given pre-stretch and frequency.

## Measurement And Characterization

### Materials for DEAs

A VHB membrane from 3M, the main member of dielectric elastomer, can produce 100–2,200% actuation strain under the combined actuation of voltage and mechanical force (Pelrine et al., 2000; An et al., 2015). The deformation scale of DE is significantly larger than piezoelectric and shape-memory materials. The maximum output stress and the optimal electromechanical conversion efficiency are comparable to human muscle tissue. Therefore, VHB-based DE actuators are also known as artificial muscle materials, and they are widely used in soft actuators especially in cases where large deformations are required. In this paper, the VHB 4,910 membrane was employed as DE material in the experiment.

### Fabrication of DEAs and Experimental Setup

**Figure 1:**
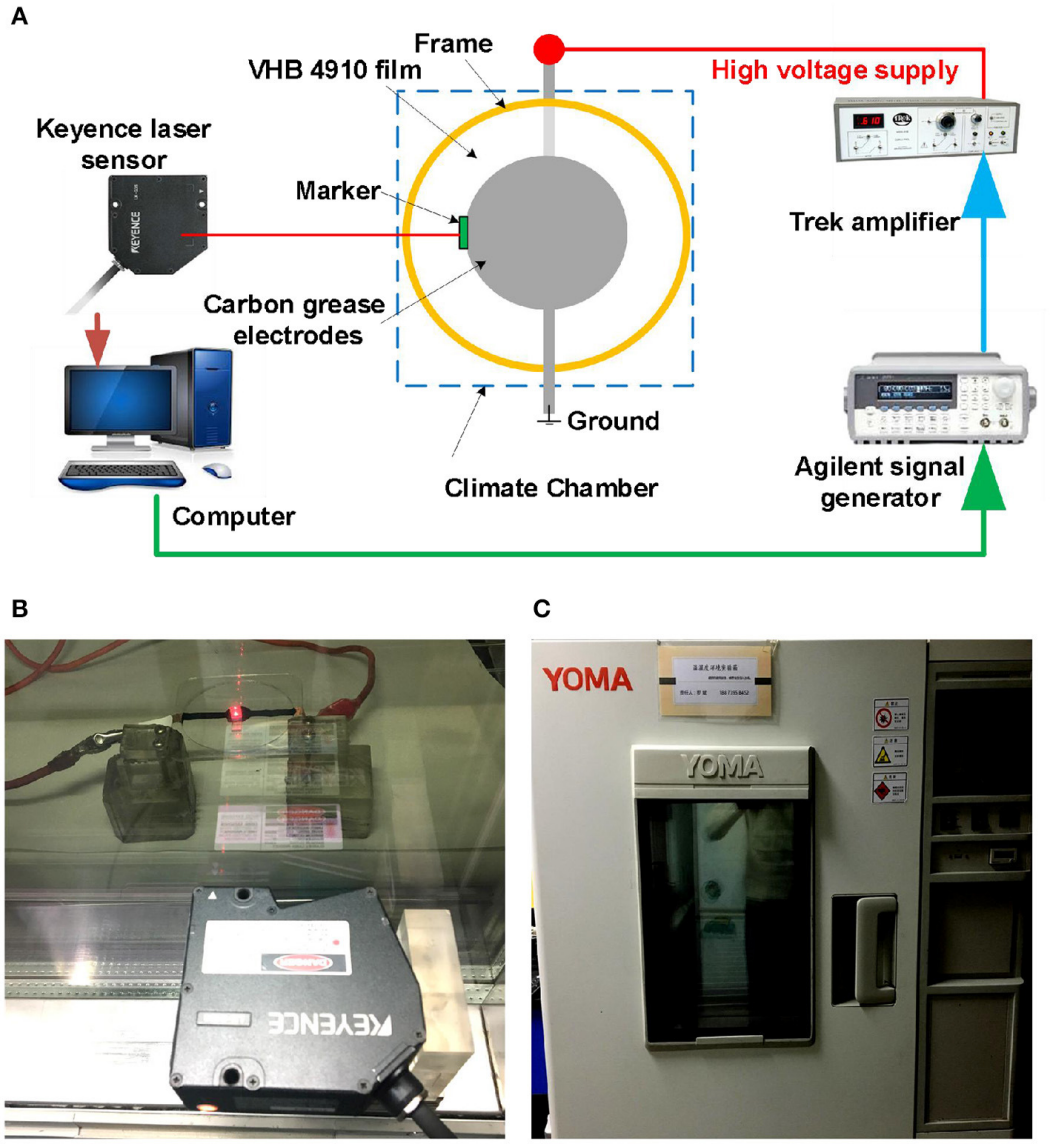
Experimental setup for dynamic breakdown tests of DEAs. **(A)** The schematic diagram of the experimental platform. (**B)** Photograph of a DEA under dynamic voltage excitation. **(C)** The climate chamber with constant humidity and temperature.

In this article, VHB 4,910 (3M Company) was utilized as the DEA material due to its outstanding large deformability. The VHB 4,910 membrane was equal-biaxially pre-stretched and fixed between a pair of 60 mm diameter supporting ring frames. VHB 4,910 film has an initial thickness of 1 mm, and it was stretched equal-biaxially to the pre-stretch ratios 4 × 4; the thickness was thus reduced to 0.0625 mm. Carbon grease (No. 846, MG Chemicals) was adopted as electrodes thanks to the extraordinary extensibility, which were coated in the middle area of both surfaces, forming a circular configuration with a diameter of 10 mm. A voltage amplifier (Model 610E, Trek) was utilized to produce the high voltage for actuation, and a signal generator (6811B, Agilent) was used to generate the cyclic voltage signal. A laser-based displacement sensor (LK-G150/G10, Kenyence) was employed to measure the electromechanical displacement of VHB film, by attaching a lightweight marker on the edge of the electro-active area, as shown in Figure 1A. The environmental temperature and humidity were controlled and tuned by a climate chamber (MHL-02S/12, Yoma), as displayed in Figure 1C. The photograph of DEA under dynamic excitation is shown in Figure 1B.

### Methods and Parameter Settings

To eliminate the interference of environmental factors, we first set the environmental temperature to 20°C and humidity to 50% through the climate chamber. The applied cyclic voltages has a peak voltage Φ_*p*_ and a valley voltage Φ_*v*_. The voltage amplitude, namely, the alternating component, is calculated as Φ_*AC*_ = (Φ_*p*_−Φ_*v*_)/2, and the direct component is Φ_*DC*_ = (Φ_*p*_+Φ_*v*_)/2. The alternating/direct component ratio (A/D ratio) is defined as β = Φ_*AC*_/Φ_*DC*_.

In order to explore the changing trend between dynamic deformation and peak voltage, deformation tests were conducted under sinusoidal voltage excitation with the following parameters: A/D ratio β = 1, frequency *f* = 2 Hz, and peak voltages 4, 6, and 8 kV. It is easy to deduce that the voltage amplitude equals half of the peak voltage when the A/D ratio is set to β = 1. The total actuation time was set to 200 s.

**Figure 2:**
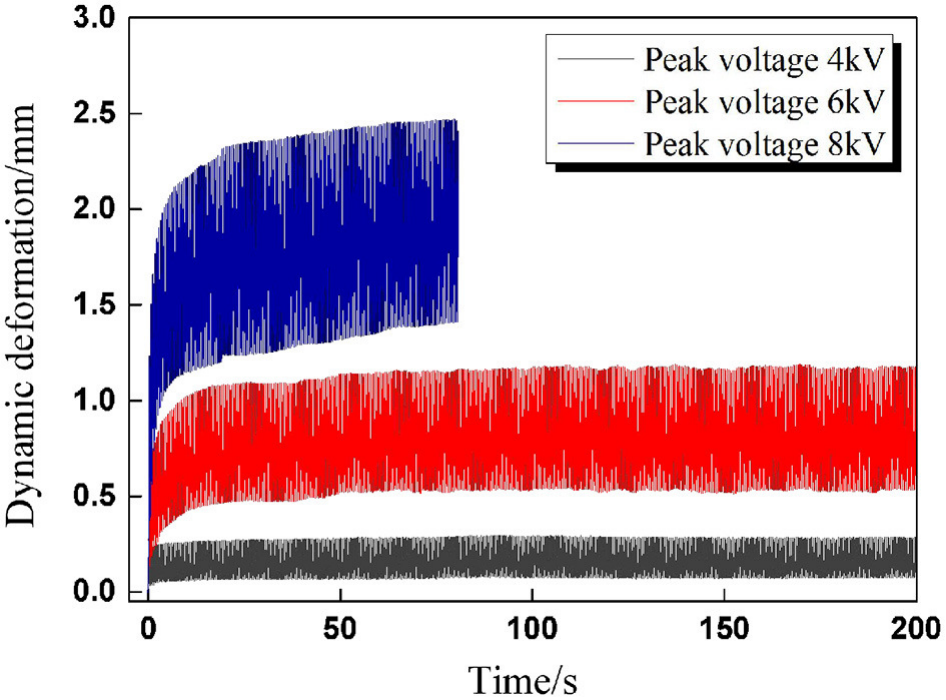
The dynamic deformation curves of DEAs under different voltage amplitudes.

The experimental results are plotted in Figure 2, and it is clear that the dynamic deformation of the DEAs rises along with the voltage amplitude; meanwhile, the viscoelastic drifts significantly with voltage amplitude, implying the reduced dynamic deformation stability. When the peak voltage is set to 8 kV, the DEA suffers premature breakdown within 200 s. In the former investigation of DEA breakdown under static excitation (Pei et al., 2004), the critical breakdown voltage is about 6.8 kV with pre-stretch 4 × 4. For a given cyclic voltage, the effective DC voltage (namely the RMS voltage Φ_*RMS*_) denotes the level of DC voltage that will produce the same energy as a cyclic voltage waveform, which is defined as follows:

(1) $$\begin{array}{l} \Phi_{RMS}=\sqrt{\frac{1}{T}\int_{0}^{T}f^{2}\left(t\right)dt} \end{array}$$

It can be calculated that when the peak voltage is 8 kV, the corresponding RMS value for sinusoidal voltage is about 4.9 kV, which is much lower than the static breakdown voltage 6.8 kV. In the subsequent dynamic breakdown tests, the RMS value of cyclic voltage should be set by the static breakdown voltage. Here, we set the change range of the ratio of RMS voltage to static breakdown voltage to 0.5~0.8, and thus the peak voltages Φ_*p*_ are determined accordingly for both sinusoidal and rectangular voltages.

A variable-controlling approach was utilized in the systematic experimental study of the dynamic breakdown behavior of DEAs. First, the effect of voltage frequency on dynamic breakdown behavior was experimentally studied. The dynamic breakdown tests were conducted under sinusoidal voltages at seven frequencies (*f* = 0.1, 0.25, 0.5, 1, 2, 5, and 10 Hz) with the peak voltage of 8 kV and the A/D Ratioβ = 1. The critical voltages and maximum cycle numbers at the breakdown were recorded. Another series of breakdown tests were carried out under rectangular voltages with the same frequencies to declare the effect of the voltage waveform.

After that, new dynamic breakdown tests were carried out to explore the influence rule of voltage amplitudes. In this part, we first set the sinusoidal voltage frequency to *f* = 2 Hz, and the A/D ratio remained the same. Seven peak voltages were selected (6, 6.5, 7, 7.5, 8, 8.5, and 9 kV). Then, another series of contrast experiments were performed under rectangular voltages with seven peak voltages under the condition of equal RMS value.

For each group of breakdown tests, 20 samples were tested to ensure the reliability of the results and obtain the statistical characteristics of dynamic breakdown behavior.

Finally, inspired by the experimental results obtained and relevant research done by other researchers, qualitative discussion on underlying mechanisms of dynamic breakdown was expressed. A power-law empirical equation was presented to characterize the relationship between maximum cycle number and nominal electrostatic stress for a given pre-stretch 4 × 4 and voltage frequency *f* = 2 Hz. Corresponding parameters were calculated based on the former test results by curve-fitting.

## Results And Discussions

### Impact of Voltage Frequency

#### Under Sinusoidal Voltages

In the first series of dynamic breakdown tests to reveal the impact of voltage frequency, sinusoidal voltages with different frequencies were first subjected to DEAs (0.1, 0.25, 0.5, 1, 2, 5, and 10 Hz).

**Figure 3:**
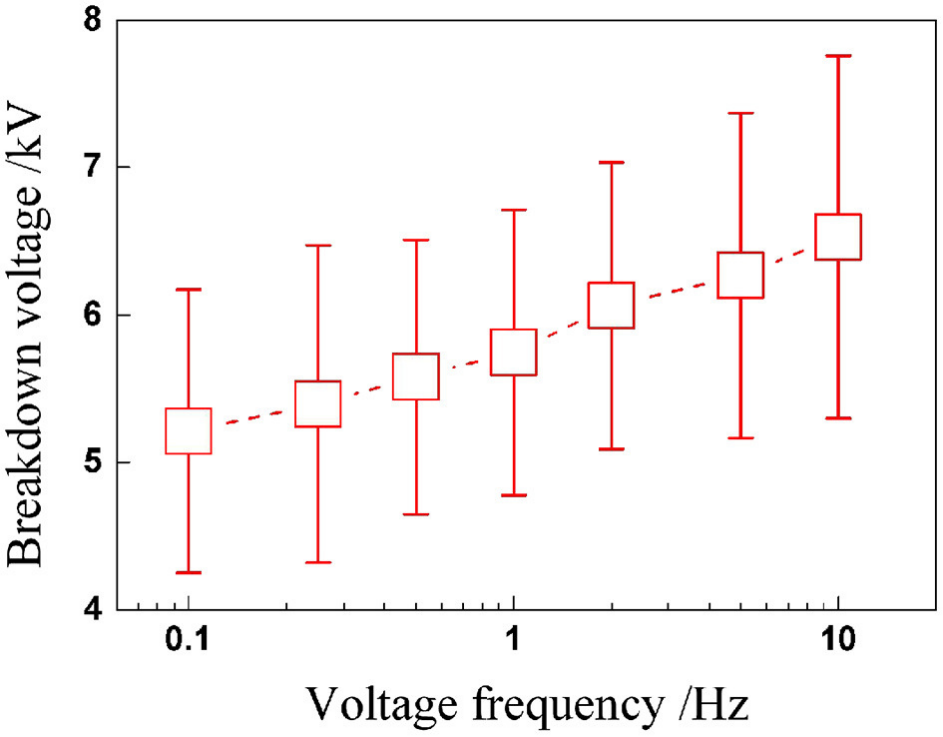
Critical voltages at breakdown under sinusoidal voltage with different frequencies.

Similar to the study on the quasi-static breakdown, we first concentrated on the critical voltage of breakdown. Figure 3 shows the experimental results: we can tell that the transient voltages at breakdown seem to be scattered, and it could be any point within the cycle. Yet, there is a slowly rising trend with voltage frequency. Unlike static breakdown under constant voltage excitation, in dynamic breakdown under cyclic voltage excitation, the maximum cycle numbers before breakdown are more important in predicting the dynamic performance of DEA.

**Figure 4:**
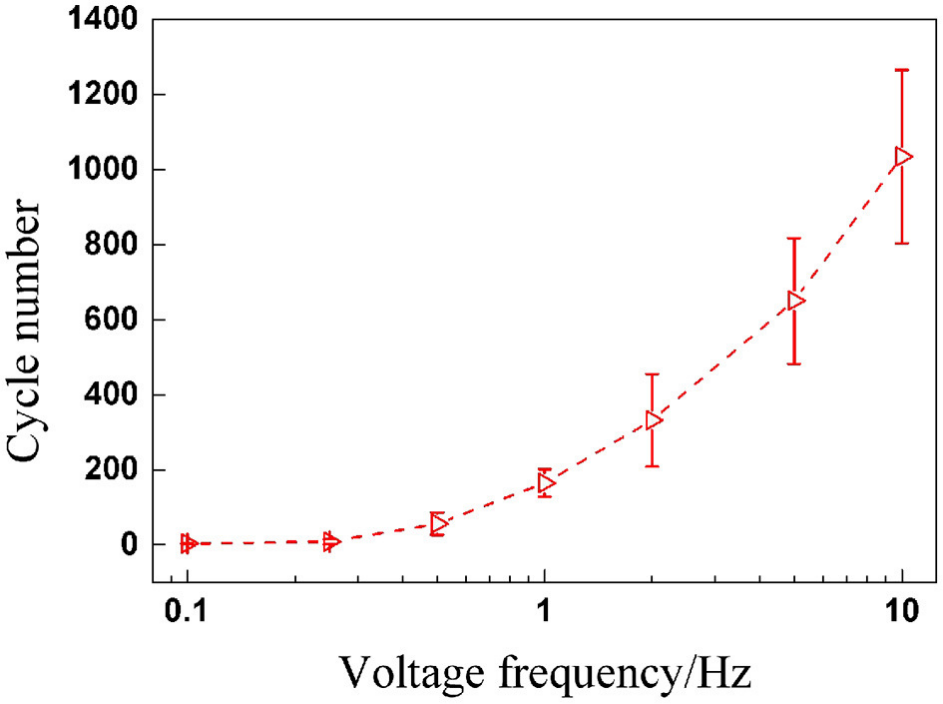
Maximum cycle numbers before breakdown under sinusoidal voltage with different frequencies.

When a DEA undergoes alternating deformation under cyclic voltages, endurance ability is definitely a performance parameter we are concerned with. Therefore, the total endurance cycle number before breakdown is worth paying attention to. Subsequently, the relationship between maximum cycle number and voltage frequency is plotted in Figure 4, which indicates that the maximum cycle number of DEAs increases nonlinearly and significantly with the voltage frequency. The underlying reason is mainly due to the strong viscoelasticity of the VHB 4,910 membrane. When the frequency rises, the voltage is altered more rapidly, leaving less time for the VHB membrane to deform in response; the maximum value for electromechanical deformation therefore declines with voltage frequency. The deformation response of the membrane can not exactly follow the rapid change of the voltage excitation. This means, although the peak value of the voltage remains the same, the internal electric field strength is greater under a low-frequency voltage excitation than that under a high-voltage frequency. So under a low frequency (with higher electric field strength), the VHB membrane is more prone to breakdown than under a high frequency. Thus maximum cycle numbers show a rising tendency with voltage frequency.

#### RMS Value of Cyclic Voltages

**Figure 5:**
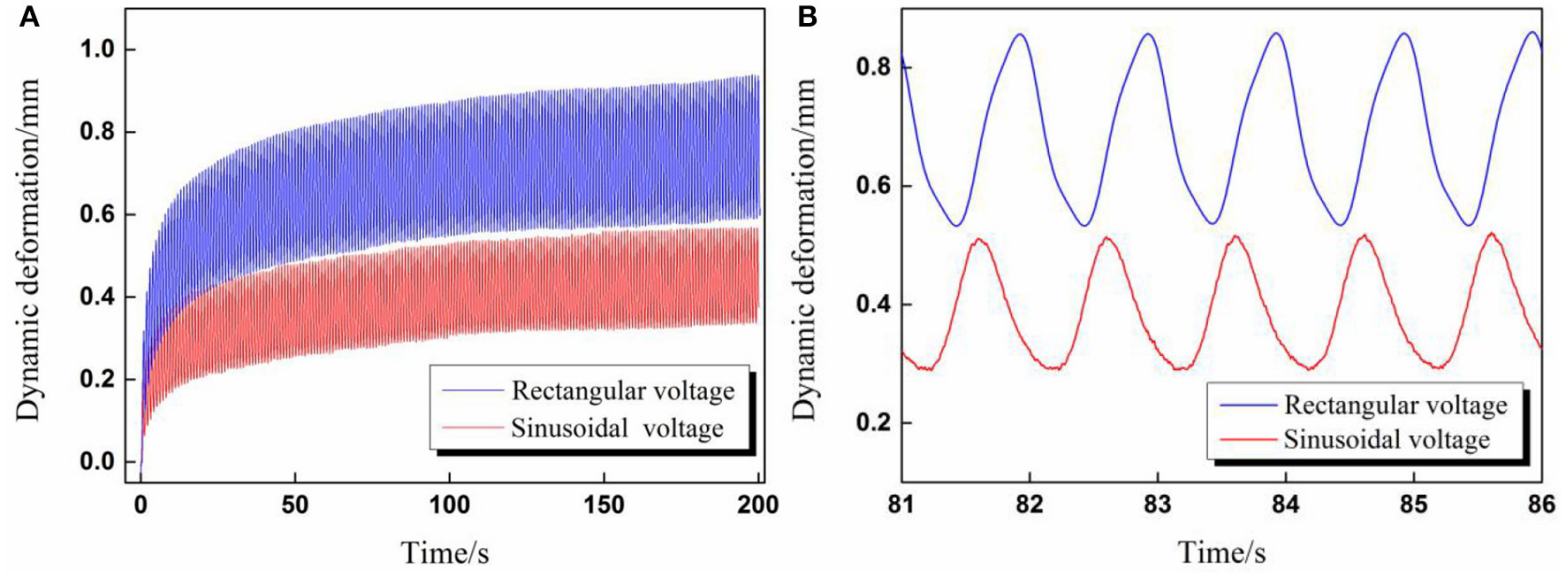
**(A)** The overall comparison diagram of dynamic deformation curves excited by rectangular and sinusoidal voltage; **(B)** Partial enlarged view of dynamic deformation curves for five cycles (Voltage amplitude 6 kV, voltage frequency 1 Hz, A/D ratio β = 1).

In the dynamic deformation tests, we have found that the deformation scale under rectangular voltage excitation was clearly larger than that under sinusoidal voltage excitation, although the voltage parameters were set exactly the same, as shown in Figure 5.

The aforementioned phenomenon can be explained by the different root mean square value Φ_*RMS*_ of applied cyclic voltages. According to Equation (1), the RMS values Φ_*RMS*_ in Figure 5 were calculated, the RMS value for sinusoidal voltage equaled 3.35 kV, and the RMS value for rectangular voltage equaled 4.24 kV. To verify the above explanation, validation experiments were carried out in which the voltage parameters for rectangular voltage remained the same while the amplitude of the sinusoidal voltage increased to ensure both waveforms have the same RMS value Φ_*RMS*_ = 4.24 kV. In addition, a step voltage with the same value 4.24 kV was also applied to DEA to give a reference deformation curve.

**Figure 6:**
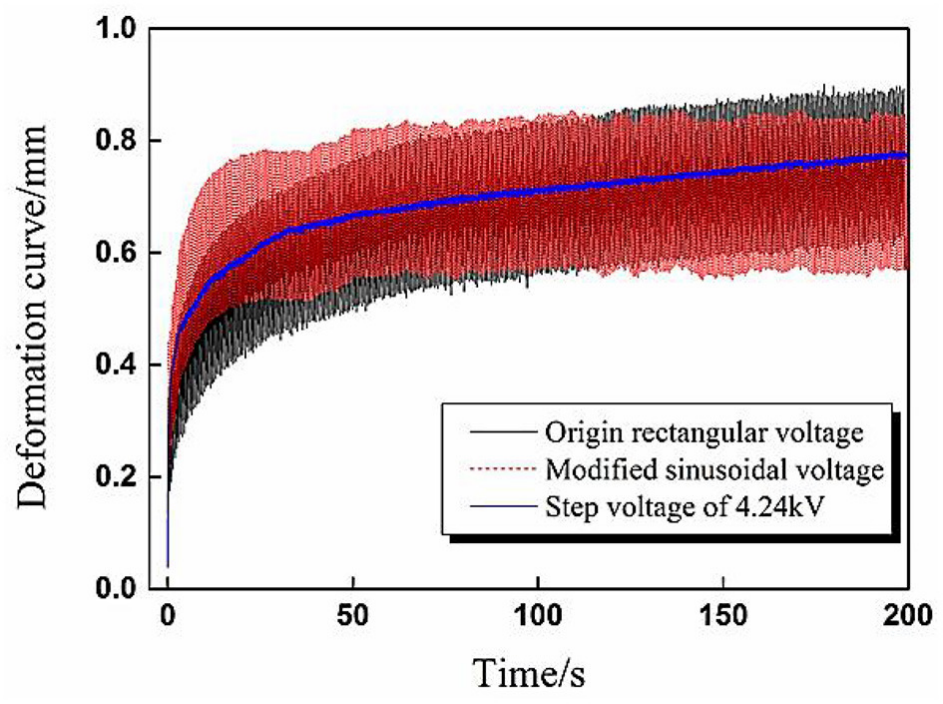
Verification experiment results of dynamic deformation under sinusoidal and rectangular voltages with the same effective voltage value 4.24 kV.

The experimental results were presented in Figure 6, where it is evident that both of the dynamic deformation curves under two cyclic voltages have good coherence with the reference curve.

#### Under Rectangular Voltages

**Figure 7:**
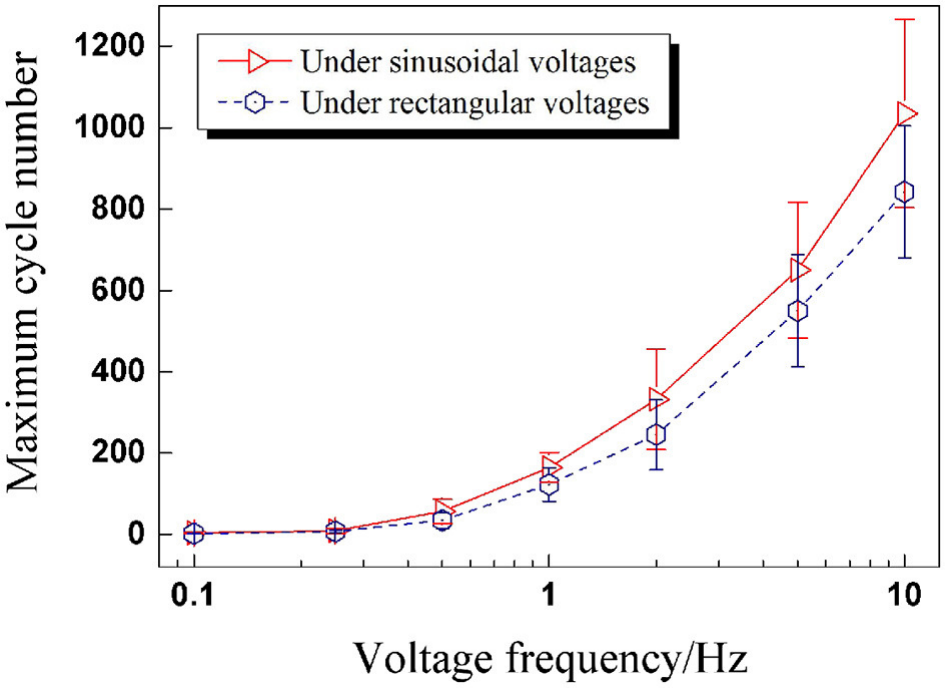
The maximum cycle numbers of DEAs under sinusoidal and rectangular voltages with different frequencies (the effective voltage value is 4.24 kV).

In this section, DEAs were subjected to rectangular voltages with the same seven frequencies to compare with the results of sinusoidal voltages. Inspired by the analysis in Section RMS Value of Cyclic Voltages, the RMS value seems to be the key parameter to determine the contained electrical energy of a cyclic voltage. In Section Under Sinusoidal Voltages, the applied sinusoidal voltages' peak values were set to 8kV. While in this part, the peak value of rectangular voltage was calculated to be 7 kV according to Equation (1). From the perspective of the application, service life was one of the most important performance parameters of DEA we were concerned with. Therefore the maximum cycle number is a key index to evaluate the dynamic performance of DEA under cyclic excitation. The maximum cycle numbers under different rectangular voltage frequencies were recorded and plotted in Figure 7 to compare with the results of sinusoidal voltages.

Figure 7 illustrates that the maximum cycle numbers for both waveforms are approximately the same when the voltage frequency is low. As the frequency goes up, the maximum cycle numbers for rectangular voltages get less than those of sinusoidal voltages, and the difference between the two maximum cycle numbers shows an increasing tendency. What is more, the maximum cycle numbers under sinusoidal voltages are more dispersed than under rectangular voltages.

### Impact of Voltage Amplitude

#### Under Sinusoidal Voltages

**Figure 8:**
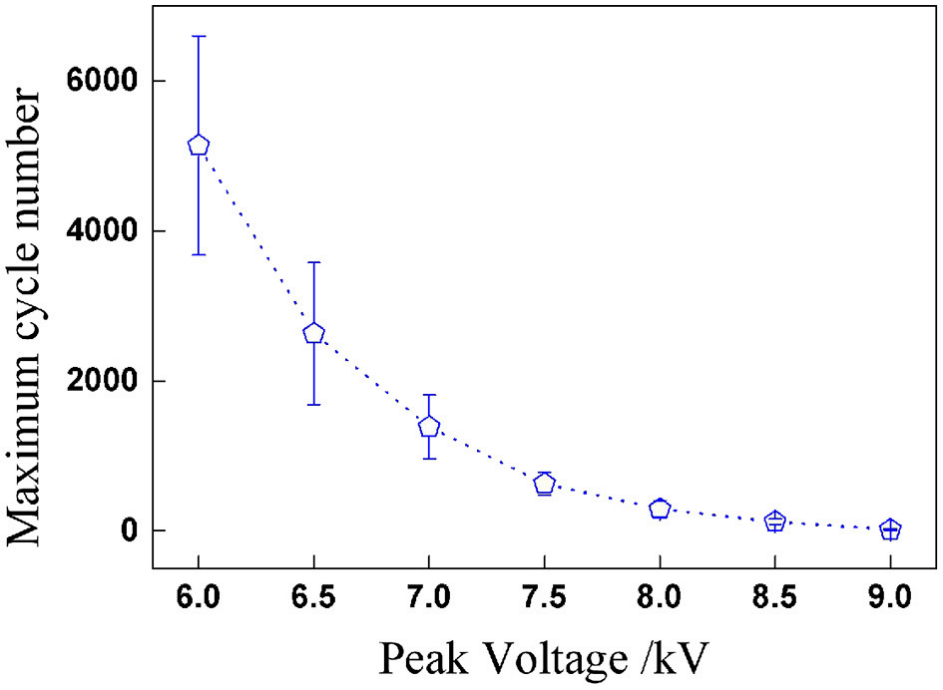
Maximum cycle numbers before breakdown under sinusoidal voltage with different peak voltages.

In the second series of dynamic breakdown tests, the influence of peak voltage was firstly studied under sinusoidal voltages. Figure 8 plots the relationship between peak voltage and maximum cycle number; it can be concluded that the maximum cycle number declines nonlinearly with the peak voltage. When the peak voltage increases, the RMS value of applied sinusoidal voltage rises as well, which means more electrical energy is applied into the actuator, and the reduction of the maximum cycle number is thus understandable. The dispersion of the maximum cycle number decreases with the increasing peak voltage.

#### Under Rectangular Voltages

**Figure 9:**
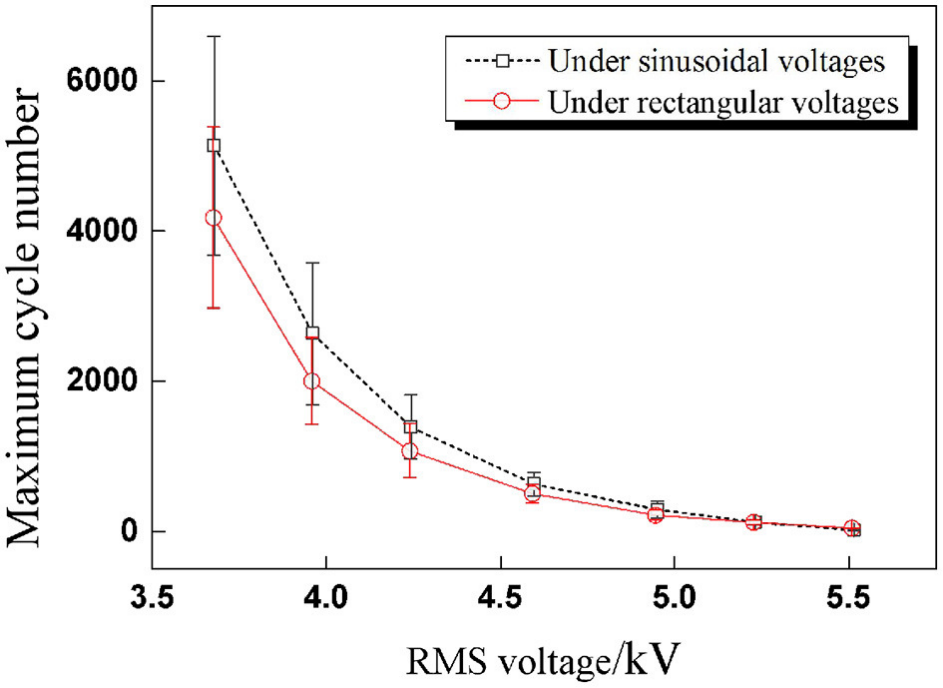
Comparison results of maximum cycle number under sinusoidal and rectangular voltages with seven RMS voltages (the effective voltage values are the same for each amplitude).

**Figure 10:**
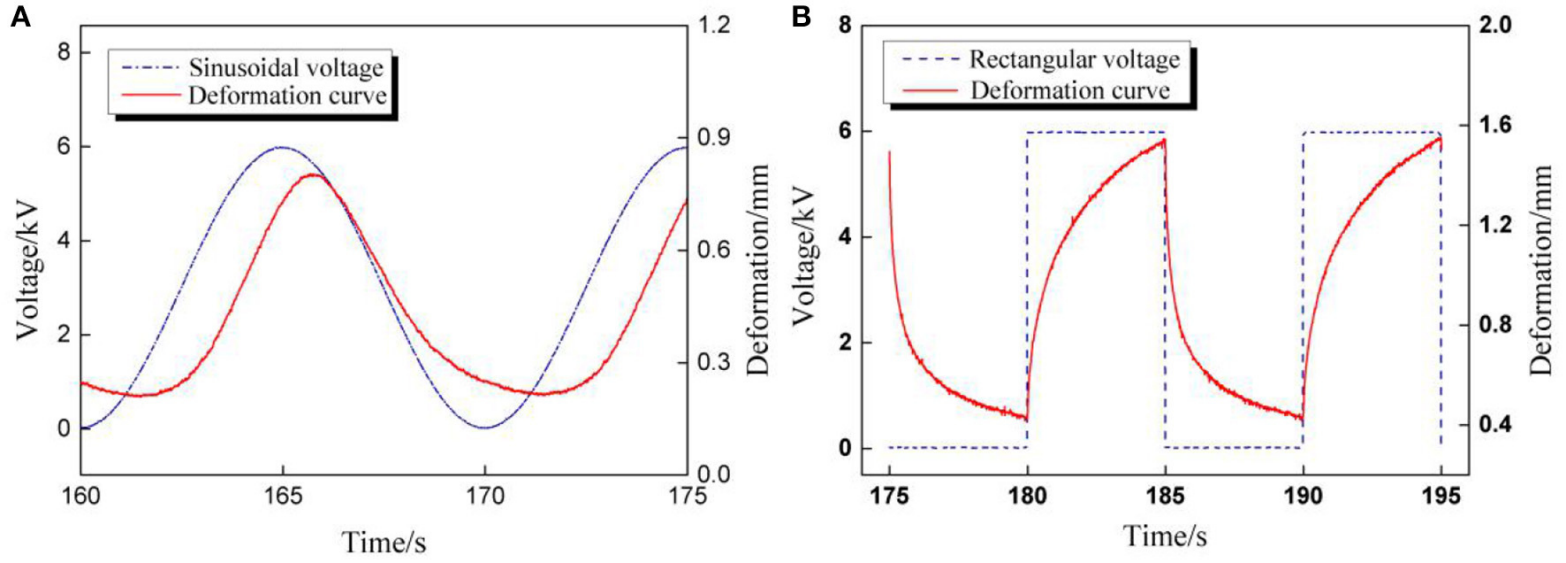
The comparisons of voltage and deformation curves excited by cyclic voltages. **(A)** Sinusoidal voltage; **(B)** Rectangular voltage.

The impact of voltage amplitude under rectangular voltage excitation was investigated subsequently. Since the RMS voltage is the parameter with which to evaluate the electrical energy of cyclic voltages, the peak values of rectangular voltage were calculated according to the sinusoidal peak values in part RMS Value of Cyclic Voltages under the condition of the same RMS value: 7.8,7.4,7,6.5,6,5.6, and 5.2 kV. For each pair of testing peak voltages, the RMS values were the same. The relationship between RMS voltages and maximum cycle number under two cyclic voltages is concluded in Figure 9. It is clear that the maximum cycle number of DEAs under sinusoidal voltage is higher than under rectangular voltages. This is mainly due to the sudden change in the applied voltage under rectangular excitation, which will lead to a sudden boost in deformation and result in a sharp change in the internal electric field strength. Yet, the deformation and electric field are strengthened under sinusoidal voltage change consecutively, as shown in Figure 10. Therefore, the durability and stability of DEAs stimulated by sinusoidal voltages are better than that of rectangular voltages, especially at higher voltage frequencies.

Currently, there is no theoretical model for the breakdown behavior of DEA under cyclic voltage excitation, and the reason is that the dynamic actuation process is far more complicated than DC voltage excitation. There are more influence parameters in cyclic excitation than DC excitation. For example, Chen et al. explored the dynamic performance of a dielectric elastomer balloon actuator, which is driven by high-pressure, and the periodic voltage applied. Snap-through instability occurs when a dielectric elastomer balloon is excited with superharmonic, harmonic, or subharmonic resonance frequency. While in this paper, dynamic breakdown behavior is investigated barely under very low frequencies, thus there is no sight of snap-through instability. In future investigations into the dynamic breakdown, the voltage frequency range shall be extended to harmonic or even superharmonic resonance frequency, and the relationship between snap-through instability and dynamic breakdown will be revealed then. Based on the test results presented in this paper and relevant research work done by others, the underlying mechanisms of DEA dynamic breakdown may be explained through the following aspects.

(a) Electrode migration: the electrode utilized in the DEA sample is carbon grease, which has good extensibility and flow-ability compared to the metal electrode. As DEA is excited by cyclic voltages, the membrane will deform alternatively along with electrodes. The adhesion and inertia forces coexist in carbon grease, it is possible that carbon grease may migrate during the actuation. This will cause an abrupt change of both charge density and internal electrostatic stress and then lead to premature breakdown.

(b) Electrode degradation: when a carbon grease electrode is exposed to the air during a long-term actuation, its performance parameters, such as shear modulus and conductivity, will vary with time. Electrode degradation is a nonlinear time-varying factor in DEA actuation. The coupling of electrode degradation and electromechanical deformation process has a combined effect on the dynamic breakdown of DEA.

(c) Viscoelasticity of VHB 4,910: the electromechanical deformation is strongly affected by viscoelastic characteristics. There is an inevitable phase delay between the voltage signal and deformation signal due to the viscoelasticity of the VHB membrane. Phase lag increases with voltage frequency, while the maximum value for alternating deformation declines with frequency. Thus, maximum cycle numbers show a rising tendency with voltage frequency.

In summary, DEA dynamic breakdown is probably a comprehensive consequence of the abovementioned effect, and a thorough and systematic model for DEA dynamic breakdown is still lacking.

### The Prediction of Maximum Cycle Number

From the dynamic experiments conducted in the former sections, it is apparent that voltage frequency and amplitude have an appreciable impact on the dynamic breakdown of DEAs. Although the onset of breakdown excited by both quasi-static and cyclic voltages is usually accompanied by ruptures and sparks, the underlying mechanism and process are not quite the same. Under a quasi-static excitation condition, we mainly focus on the change tendency of critical breakdown strength/voltage. However, when actuated by cyclic voltages, more attention should be paid to the maximum cycle number since endurance ability becomes a major parameter to evaluate the performance of DEAs.

In Figure 9, the changing rule of RMS voltage and maximum cycle number is similar to the trend of inverse proportion function, which is a power-law function. In the prediction of maximum cycle number, firstly, the nominal electrostatic stress $\bar{\sigma_{NOM}}$ is defined through RMS voltage and calculated according to Equation (2), and then the relationship between the nominal electrostatic stress and the maximum cycle number of DEAs can be expressed through a power-law empirical equation as shown in Equation (3).

(2) $$\begin{array}{l} \bar{\sigma_{NOM}}=\frac{1}{2}\varepsilon {\bar{E}}^{2}-\frac{1}{2}\varepsilon {\left[\frac{\phi_{RMS}}{H}\right]}^{2}\lambda_{p}^{4} \end{array}$$

where ε is the dielectric constant of VHB 4910, $\bar{E}$ is the effective electric field strength determined by the RMS value of the cyclic voltage, *H* is the initial thickness of VHB 4,910 membrane, and λ_*p*_ is the pre-stretch ratio of DEA (in this paper λ_*p*_ = 4).

(3) $$\begin{array}{r} N=\text{a}\cdot {\left(\bar{\sigma_{\text{NOM}}}\right)}^{\text{b}} \end{array}$$

**Figure 11:**
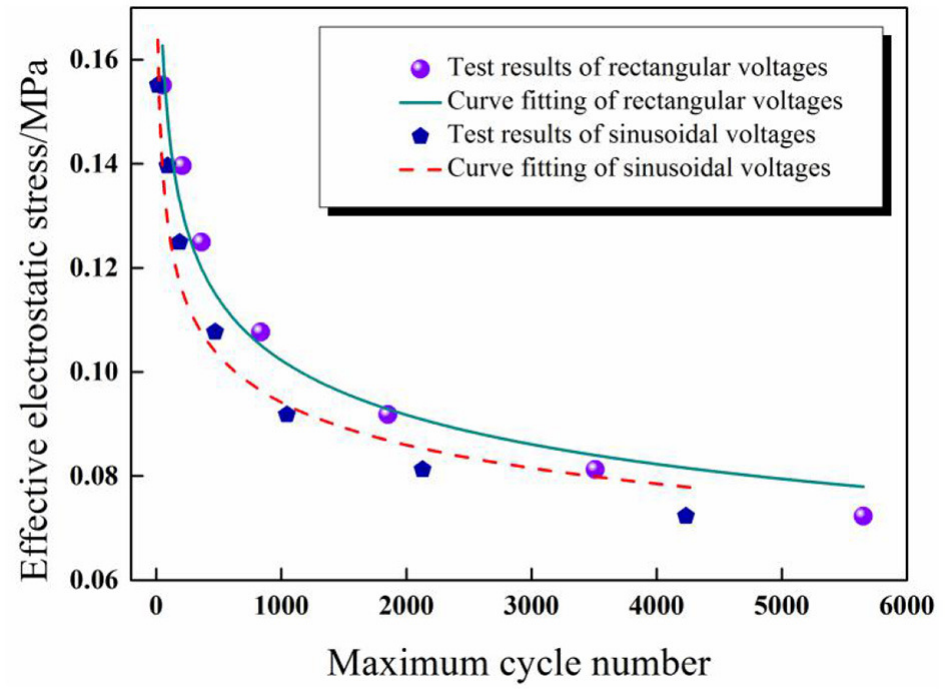
Comparison diagram of the fitting results and experimental results of maximum cycle numbers under sinusoidal and rectangular voltage excitation.

Figure 11 Comparison diagram of the fitting results and experimental results of maximum cycle numbers under sinusoidal and rectangular voltage excitation.

The experimental results were substituted into the power-law empirical Equation (3) first, and then data fitting was realized through Origin software. Figure 11 shows the test results can be fitted by the proposed power-law formula under different waveforms for a given pre-stretch and frequency, which indicates that the power-law equation is applicable to the prediction of the service life of DEAs under cyclic voltage actuation. The corresponding fitting results are as follows: *a* = 4.9 × 10^−4^, *b* = –6.37 for sinusoidal voltage and *a'*= 1.53 × 10^−5^, *b'* = –7.62 for rectangular voltage.

## Closing Remarks

In this paper, the dynamic breakdown behavior of circular DEAs made from the VHB 4,910 membrane was experimentally and systematically studied. The DEAs were subjected to both sinusoidal and rectangular voltages. The impact of voltage parameters (such as frequency and amplitude) on the dynamic breakdown of DEAs was investigated. Unlike quasi-static breakdown behavior, the maximum cycle number at breakdown is a key estimation performance when DEA is stimulated by cyclic voltage. The increase of voltage frequency will lead to a growth in the maximum cycle number, while the increase of voltage amplitude will cause an opposite trend. The durability of DEAs under sinusoidal voltage seems better than that of rectangular voltage due to the continuous change in deformation and electric field strength. At last, a power-law empirical equation was proposed to predict the maximum cycle number of DEAs under cyclic voltage excitation. The study presented in this paper offers a new way to predict the breakdown performance of DEA under cyclic voltage.

## Data Availability Statement

The original contributions presented in the study are included in the article/supplementary material, further inquiries can be directed to the corresponding author/s.

## Author Contributions

In this work, BL first proposed the research thinking and approach of the paper. The experiment platform was set up by XL, and the experiment investigation was done by YX. Some important material parameters were provided and experiment results were analyzed by WS. The experiment data were collected and fitted by ZZ. The first draft of the manuscript was written by XL and YX. SG made some important revisions to the paper. And finally all the participants approved the final version of the paper to be submitted.

## Conflict of Interest

The authors declare that the research was conducted in the absence of any commercial or financial relationships that could be construed as a potential conflict of interest.
